# Supplementary material for: The interactome of intact mitochondria by cross-linking mass spectrometry provides evidence for coexisting respiratory supercomplexes
Source: Mol Cell Proteomics. 2017 Dec 8;17(2):216–32. doi: 10.1074/mcp.RA117.000470 (PMC5795388; doi:10.1074/mcp.RA117.000470)
Supplement: Supplemental Data [file supp_RA117.000470_133922_0_supp_23978_fzffwf.zip › spectra_annotation/mito_DR_spectra_annotation/289-2-19-1-14-1.pdf]

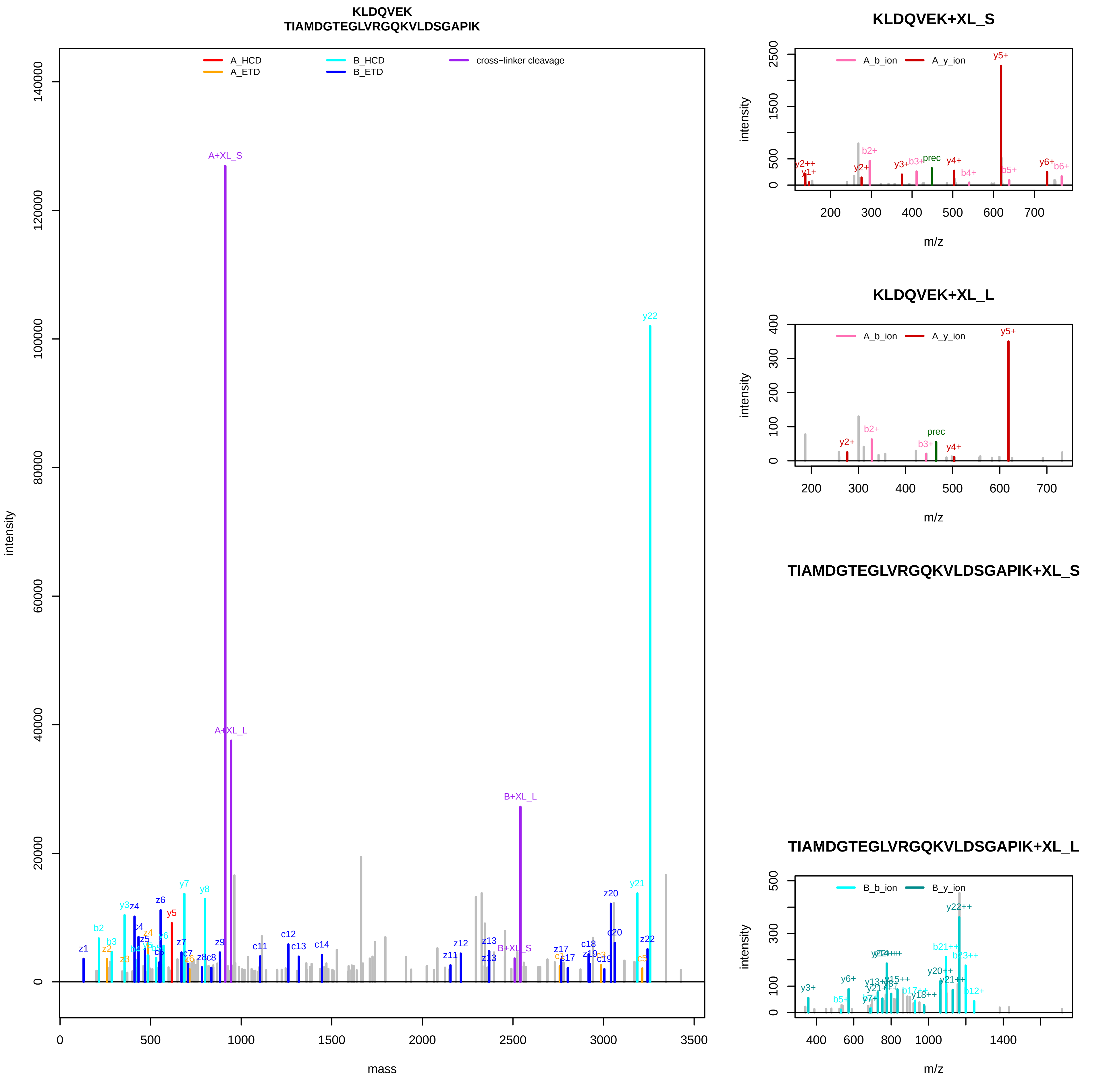
KLDQVEK
TIAMDGTEGLVRGQKVLDSGAPIK
A_HCD
A_ETD
B_HCD
B_ETD
cross−linker cleavage
A+XL_S
A+XL_L
B+XL_L
B+XL_S
y22
y21
z20
c20
z22
y7
y8
y5
z6
y3
z4
b2
b3
z1
z2
z3
c4
z5
c5
z7
c7
z8
c8
z9
c11
c12
c13
c14
z11
z12
z13
z17
c17
c18
z19
c19
intensity
mass
KLDQVEK+XL_S
A_b_ion
A_y_ion
y5+
b2+
y2++
y1+
y2+
y3+
b3+
prec
y4+
b4+
b5+
y6+
b6+
m/z
KLDQVEK+XL_L
TIAMDGTEGLVRGQKVLDSGAPIK+XL_S
TIAMDGTEGLVRGQKVLDSGAPIK+XL_L
B_b_ion
B_y_ion
y22++
b21++
b23++
y20++
y21++
y3+
b5+
y6+
y7+
y13++
y15++
y18++
b12+
